# Transcriptome-wide study revealed m6A and miRNA regulation of embryonic breast muscle development in Wenchang chickens

**DOI:** 10.3389/fvets.2022.934728

**Published:** 2022-07-26

**Authors:** Lihong Gu, Qicheng Jiang, Youyi Chen, Xinli Zheng, Hailong Zhou, Tieshan Xu

**Affiliations:** ^1^Institute of Animal Science and Veterinary Medicine, Hainan Academy of Agricultural Sciences, Haikou, China; ^2^School of Life Science, Hainan University, Haikou, China; ^3^Wuzhishan Animal Science and Veterinary Medicine and Fishery Service Center, Wuzhishan Agricultural and Rural Bureau, Wuzhishan, China; ^4^Tropical Crops Genetic Resources Institute, Chinese Academy of Tropical Agricultural Sciences, Haikou, China

**Keywords:** m6A, Wenchang chickens, miRNA, breast muscle, muscle development, fat deposition

## Abstract

*N*^6^-Methyladenosine (m6A) modification has been shown to play important role in skeletal muscle development. Wenchang chickens are commonly used as a high-quality animal model in researching meat quality. However, there have been no previous reports regarding the profile of m6A and its function in the embryonic breast muscle development of Wenchang chickens. In this paper, we identified different developmental stages of breast muscle in Wenchang chickens and performed m6A sequencing and miRNA sequencing in the breast muscle of embryos. Embryo breast muscles were weighed and stained with hematoxylin–eosin after hatching. We found that myofibers grew fast on the 10^th^ day after hatching (E10) and seldom proliferated beyond the 19^th^ day after hatching (E19). A total of 6,774 differentially methylated genes (DMGs) were identified between E10 and E19. For RNA-seq data, we found 5,586 differentially expressed genes (DEGs). After overlapping DEGs and DMGs, we recorded 651 shared genes (DEMGs). Subsequently, we performed miRNA-seq analysis and obtained 495 differentially expressed miRNAs (DEMs). Then, we overlapped DEMGs and the target genes of DEMs and obtained 72 overlapped genes (called miRNA-m6A-genes in this study). GO and KEGG results showed DEMGs enriched in many muscle development-related pathways. Furthermore, we chose WNT7B, a key regulator of skeletal muscle development, to perform IGV visualization analysis and found that the m6A levels on the WNT7B gene between E10 and E19 were significantly different. In conclusion, we found that miRNAs, in conjunction with m6A modification, played a key role in the embryonic breast muscle development of Wenchang chickens. The results of this paper offer a theoretical basis for the study of m6A function in muscle development and fat deposition of Wenchang chickens.

## Introduction

Unlike DNA modification, RNA modification is more complex and diverse. More than 150 types of RNA modifications are currently known, most of which occur after transcription (1). RNA modifications have an important impact on the diversity of RNA structure, type, and function. *N*^6^-Methyladenosine (m6A) modification is the most common post-transcriptional RNA modification in eukaryotes and the most common internal modification in messenger RNA (mRNA) (2).

m6A modification is driven by the m6A modified enzyme system, which can be divided into three categories: writers, erasers, and readers. Among them, the writers, also called m6A methyltransferases, include METTL3/14, WTAP, and KIAA1429; their main function is to catalyze m6A modification of adenylate on mRNA. The erasers are also called m6A demethylases; their main function is to demethylate the modification of bases that have undergone m6A modification. Currently, the main m6A demethylases include two members, obesity-related protein (FTO) (3) and Alk B homologous protein 5 (ALKBH5) (4). These two demethylases lead to a reversible change in m6A modification. The readers, also called m6A binding proteins, are mainly used to identify m6A modified bases and activate downstream regulatory pathways, such as RNA degradation and miRNA processing. The m6A readers mostly include YTH family proteins, such as YTHDC1, YTHDF1, and YTHDF2. YTHDC1 interacts with the splicing factors SRSF3 and SRSF10 to affect transcript expression (5). YTHDF1 can be complexed with translation initiation action, thereby promoting the translation of m6A modified RNA (6). YTHDF2 can mediate the degradation of m6A modified RNA (7).

Increasing numbers of research studies, based on m6A modification, have found it to play important regulatory role in many physiological processes. Hao et al. showed that METTL3-mediated m6A modification plays an important role in biological processes such as mouse embryo development (8). Yang et al. found that RNA m6A modification is involved in the regulation of circadian rhythms in the chicken hypothalamus under both basal and chronic stress conditions (9). m6A modification also has many functions in embryonic stem cell differentiation, development of the nervous and hematopoietic systems, myogenesis, zygote formation, and embryonic development (10). The regulation of m6A methylation is integral to muscle generation during embryonic development. Meanwhile, Wang et al. (11) found that FTO is required for myogenic differentiation and suggested that the FTO-mediated mTOR-PGC-1α-mitochondrial axis plays crucial role in myogenic differentiation. Zhang et al. (12) detected the specific expression of METTL3 in the vascular system of zebrafish. It is speculated that m6A modification is closely related to blood development, and the key role of m6A methylation modification in the development of hematopoietic stem cells has been revealed for the first time. m6A is also an important regulator of muscle development. Chen et. al. (13) explored the involvement of m6A mRNA modifications in mediating muscle regulation. Dang et al. (14) identified the key genes involved in cattle muscle growth and m6A modification development by bioinformatics analysis. The results showed that the differentially expressed genes modified by m6A are mainly involved in skeletal muscle contraction, steroid biosynthesis, redox process, the PPAR pathway, and fatty acid metabolism. Finally, Yang et al. (15) used m6A-seq to analyze bovine myoblasts and myotubes and found that m6A methylation was an abundant modification in mRNA. Furthermore, using experiments, they confirmed that four genes related to myogenesis exhibited differential changes in both m6A and mRNA levels during bovine myoblast differentiation, indicating that they can be potential candidate targets for m6A regulation of skeletal myogenesis.

MicroRNA (miRNA) is a type of noncoding RNA that exists widely in eukaryotes with a length of about 18–25 nt. miRNA plays an important role in the post-transcriptional regulation of target genes and is widely involved in various biological processes including growth and development, immunity, proliferation, and apoptosis. Shen et al. found that the expression of miR-152 can affect the quality of pork (16). Jebessa et. al. (17) investigated the underlying molecular mechanisms of skeletal muscle development based on differentially expressed genes and miRNAs. Meanwhile, Li et. al. (18) summarized miRNA related to muscle development, providing a better understanding of skeletal muscle development.

The rearing of Wenchang chickens is the most economically important livestock sector in Hainan province, with a 1.78 billion dollars output value in 2020. Wenchang chickens are famous for their rough feeding resistance and heat resistance and have a higher intramuscular fat and moderate subcutaneous fat content. Our research showed that the fastest stage of embryonic breast muscle development of Wenchang chickens was E10, and growth had slowed by E19. Given that m6A methylation modification plays an important role in skeletal muscle development, we speculated that modification of m6A methylation might be a crucial regulator of the growth rate of embryonic breast muscle. Herein, we performed m6A sequencing of breast muscle on embryos at E10 and E19. We also performed miRNA-seq to explore whether some genes were regulated by m6A and miRNA. We set out to determine the m6A profile and miRNA regulation in the embryonic breast muscle of Wenchang chickens; the results of this paper offer a basis for revealing the role of m6A modification in breast muscle development.

## Methods

### Ethics approval

This experiment was performed in accordance with animal welfare principles and was conducted under protocols approved by the Chinese Universities Union for the Protection of Animals.

All chickens were obtained from the Institute of Animal Science & Veterinary Medicine, Hainan Academy of Agricultural Sciences (IASVM-HAAS, Haikou, China). Ethics approval (reference number: IASVMHAAS-AE-202016) was conferred by the animal ethics committee of IASVM-HAAS, which is responsible for animal welfare. All experimental protocols were conducted in accordance with guidelines established by the Ministry of Science and Technology (Beijing, China).

### Anatomy experiment

To identify different developmental stages of breast muscle in Wenchang chickens, we selected three eggs per day from the 8^th^ day after hatching (E8) and the 21^st^ day after hatching (E21). Embryo weight and breast muscle weight were recorded, and then, breast muscles from E8 to E21 were stripped from the bone and stained with HE staining by the same process as Gu et al. (19). Briefly, breast muscle samples of fourteen embryonic stages (E8–E21) were washed with running water and then dehydrated in a series of ethanol dilutions (75% for 4 h, 85% for 4 h, and 95% overnight) and then 100% ethanol for 2 h with two changes. Dehydrated tissues were treated with xylene three times and then embedded into paraffin blocks, trimmed, and cut to 4 μm. Paraffin ribbons were placed in a water bath at about 40 °C. Sections were mounted onto slides, air-dried for 30 min, and then dehydrated at 45 °C overnight. Sections were dewaxed with two changes of xylene for 10 min each and then hydrated with two changes of 100% ethanol for 3 min each, 95% and 80% ethanol for 1 min each, and, finally, rinsed in distilled water for 5 min. Slices were stained with hematoxylin and eosin (H&E). Sections from three samples of breast muscle were taken from each stage; five different fields were examined from each section, and pictures were taken under each field; cell counts were also performed for each field.

### Sample collection

The Wenchang chicken embryos at E10 and E19 were purchased from the breeding farm of Hainan Chuanwei Wenchang Chickens Industry Co. Ltd. Three eggs were selected at E10 and E19; embryos were removed under aseptic conditions and stripped of their breast muscles; the left and right sides for each embryo were placed into different centrifuge tubes. The centrifuge tubes were placed immediately into liquid nitrogen and subsequently brought to the laboratory for storage at−80 °C for further use. The left and right breast muscles of chicken embryos were used for m6A-seq and RNA-seq analysis, respectively. In addition, another three embryonic breast muscle samples from E10 and E19 embryos were obtained and were used for miRNA-seq analysis.

### RNA extraction and fragmentation

Total RNA was isolated and purified using TRIzol reagent (Invitrogen, Carlsbad, CA, USA) following the manufacturer's instructions. We purified Poly (A) RNA from total RNA in three steps. The first step used NanoDrop ND-1000 (NanoDrop, Wilmington, DE, USA) to quantify the RNA and purity of each sample. The next step confirmed the RNA integrity, as assessed by Bioanalyzer 2100 (Agilent, CA, USA) with RIN number >7.0, using electrophoresis with denaturing agarose gel. The final step was purifying Poly (A) RNA from 50 μg total RNA using Dynabeads Oligo (dT) 25-61005 (Thermo Fisher, CA, USA) with two rounds of purification. The fragmentation buffer was added to the purified Poly (A) mRNA for fragmentation.

### m6A immunoprecipitation, library construction, and sequencing

The fragmented RNA was divided into two parts. The first part was incubated for 2 h at 4 °C with m6A-specific antibody (No. 202003, Synaptic Systems, Germany) in IP buffer (50 mM Tris-HCl, 750 mM NaCl, and 0.5% IGEPAL CA-630). To create the cDNA, we made IP RNA reverse-transcribed with SuperScript™ II Reverse Transcriptase (Invitrogen, cat. 1896649, USA) according to the technical manual. Then, the products were used to synthesize U-labeled second-stranded DNAs with *E. coli* DNA polymerase I (NEB, cat.m0209, USA), RNase H (NEB, cat.m0297, USA), and dUTP Solution (Thermo Fisher, cat.R0133, USA). Because each adapter contains a T-base overhang, we added an A-base to the blunt ends of each strand for ligating the adapter. Single- or dual-index adapters were ligated to the fragments, and size selection was performed with AMPure XP beads. Subsequently, the heat-labile UDG enzyme (NEB, cat.m0280, USA) treatment of the U-labeled second-stranded DNAs was performed. The products were amplified with PCR under the following conditions: initial denaturation at 95 °C for 3 min; 8 cycles of denaturation at 98 °C for 15 s, annealing at 60 °C for 15 s, and extension at 72 °C for 30 s; and then final extension at 72 °C for 5 min. The average insert size for the final cDNA library was 300 ± 50 bp.

The second part served as a control to construct a conventional transcriptome sequencing library directly. The two constructed sequencing libraries, m6A-seq Library (IP) and RNA-seq Library (input), were separately subjected to high-throughput sequencing using Illumina NovaSeq™ 6000, and the sequencing mode was 150 PE.

### M6A-seq and RNA-seq data filtering

M6A-seq technology employs a co-immunoprecipitation approach in which m6A-specific antibodies are incubated with RNA fragments that are randomly interrupted (IP) and fragments that grasp the m6A methylation modification are sequenced. One control group (input) was simultaneously sequenced in parallel and used to eliminate background during grasping with methylated fragments (20). So we performed m6A-seq in concert with RNA-seq. First, the reads containing adapter contamination, low-quality bases, and undetermined bases were removed by fastp (https://github.com/OpenGene/fastp) with default parameters, and valid data were then obtained. Sequence quality of IP and input samples were also verified using fastp. The R package exomePeak 1.8 (21) provided mapped reads for IP reads and input libraries, which identifies m6A peaks with bed or bigwig format. Furthermore, we use motif analysis software MEME 1.0 to identify motifs with high reliability in the peak area and record the width, E-value, PFM, PSSM of each motif, and its total position information in each peak sequence, then performed motif prediction for each group of samples, and analyzed the differential expression results.

### m6A methylation peak screening and differential m6A analysis

The peak calling software and the R package exomePeak 1.8 were used to perform peak scanning on m6A samples and transcriptome samples to obtain the location of the peak on the genome, peak length, and the difference calculations between groups. These peaks were finally annotated using ChIPseeker 1.0.

### Reference genome alignment

Valid data were aligned onto the reference genome of chicken (*Gallus gallus*, Version: V96) using the software HISAT2 1.0. According to the genomic annotation file gtf, number and distribution of alignment to reference genome reads, reads number, and proportions in exon and intron were counted. Valid data that enabled alignment to the reference genome, in accordance with the regional information of the reference genome, could be defined as alignment to exon (exonic), intron (intronic), and intergenic (intergenic spacer region) data.

### Differentially expressed genes

StringTie 1.0 (22) was used to detect the expression levels for all mRNAs from Input libraries by calculating FPKM [total exon fragments /mapped reads (millions) × exon length (kB)]. The differentially expressed mRNAs were selected according to the criteria of |log2foldchange|≥1 and p<0.05 by R package edgeR 4.1 (https://bioconductor.org/packages/edgeR) (23).

### Conjoint analysis of DEGs and DMGs

We overlapped DEGs and DMGs to explore gene modification by m6A and differential genes that were potentially connected with breast muscles. If a DEG overlapped with one or more differentially methylated m6A peaks, it was called a differentially methylated gene (DEMG). We examined up- and down-overlapping methylated m6A sites with up- and downregulated genes; we then obtained the upregulated genes with upregulated methylated m6A sites (hyper-up), the downregulated genes with upregulated methylated m6A sites (hyper-down), the upregulated genes with downregulated methylated m6A sites (hypo-up), and the downregulated genes with hypo-methylated m6A sites (hypo-down).

### miRNA library construction, sequencing, and analysis

The experimental flow was performed following standard procedures provided by Illumina. Small RNA sequencing libraries were prepared using the TruSeq Small RNA Sample Prep Kits (Illumina, San Diego, USA). After library preparation was completed, the constructed libraries were sequenced using Illumina hiseq^2500^ with a sequencing read length of single-end 1 ×50 bp.

ACGT101-miR 4.2 (LC Sciences, Houston, Texas, USA) was used to analyze the miRNA data. First, 3'-adaptors and junk sequences were removed to obtain sequences with lengths in the range of 18–26 nt. The remaining sequences were then aligned (miRNA excluded) to the mRNA, Rfam, and Repbase databases and filtered. Valid data were then aligned to pre-miRNA and the genome for miRNA identification. The identified miRNAs were differentially analyzed, and the differential miRNAs were subjected to target gene prediction.

### Integrated analysis of m6A-seq, mRNA-seq, and miRNA-seq data

After differentially methylated m6A peaks and DEGs were obtained, we searched for DEGs to examine whether a DEG overlapped with one or more differentially methylated m6A peaks. If such an overlap was demonstrated, it was termed a differentially methylated gene (DEMG). By the analysis of miRNAs, we obtained differential miRNAs (DEMs). The targets of DEMs were then predicted. Finally, we overlapped the targets of DEMs and DEMGs and obtained the shared genes.

## Results

### Determination of developmental regularity of Wenchang chicken breast muscle

**Figure 1:**
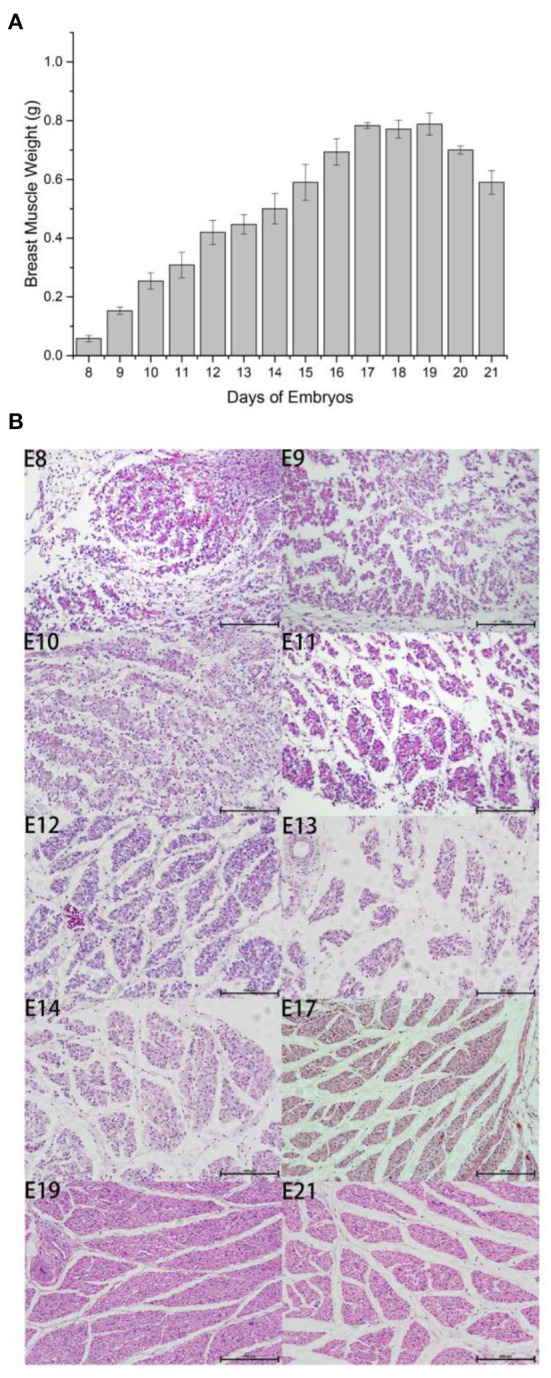
Outline of breast muscle development during the embryonic stages of Wenchang chickens. **(A)** Trend of breast muscle weights. **(B)** Embryonic breast muscle slices of Wenchang chickens. 8–14, 17, 19, and 21 represented to embryonic 8^th^ day E8–E14, E17, E19, and E21, respectively.

We measured embryo weight and breast muscle weight and performed H&E staining for Wenchang chicken embryos at stages E8–E19. The weight of the breast muscle increased gradually from E8 to E17, (Figure 1A) then plateaued after E17, and decreased during the first few days of life, which may indicate that the breast muscle was initially in a proliferative differentiation phase; then during the first few days of life, the nutrition in the muscle was consumed, and the weight of the breast muscle decreased. By counting the results and section results in Figure 1B, it can be seen that the breast muscle cells were in the most proliferative period during E8–E12, and the myofibers gradually converged to form muscle bundles after E12; however, large numbers of free myocytes remained. By E17, the myofibers were progressively larger, free myocytes were progressively fewer, myocyte fusion events were gradually reduced, and the number of myofibers was concomitantly fixed. For E10–E12, the fastest growth took place at E10 and there was a gradual decline in number between E17 and E21 because of the progressive enlargement of myofibers with cessation of proliferation. Therefore, we selected days E10 and E19 to investigate the potential regulation of m6A modification in Wenchang chicken embryonic skeletal muscle using m6A-seq technology.

### Basic information of m6A-seq and RNA-seq

For RNA-seq (input), 60.53 G raw data were obtained from breast muscles at E10 and E19, with an average of 10.09 G per sample. The raw data were filtered by fastp, and a mean of 6.94 G was obtained per sample. For m6A-seq (IP), 64.07 G raw data were obtained at E10 and E19, with a mean of 10.67G per sample. After filtering, a total of 43.02 G of valid data were obtained, with a mean of 7.17 G per sample (Supplementary Table S1).

### Alignment of valid data to the chicken reference genome

We then aligned the valid data to the chicken reference genome (Gallus gallus Version: V96) (Supplementary Table S2). Mean alignment rates were 90.83% for RNA-seq and 87.07% for m6A-seq. For m6A-seq, the majority of valid reads were aligned to exonic regions (exon) with 88.96, 87.7, and 87.67% for the three samples of E10 and 91.57, 92.96, and 92.49% for E19. Only a small proportion of the valid reads fell in intronic and intergenic regions. Similar to m6A-seq, most of the valid reads of RNA-seq aligned to exonic regions with 87.04, 87.81, and 89.07% for the three samples of E10 and 89.79, 90.51, and 89.84% for E19. In this study, most valid reads fell in exonic regions, which was consistent with the fact that the m6A-seq and RNA-seq libraries were constructed using Poly (A) RNA (Supplementary Figure S1). The results above indicate that our sequencing results were accurate.

### m6A methylation peak screening and differential m6A peak analysis

**Figure 2:**
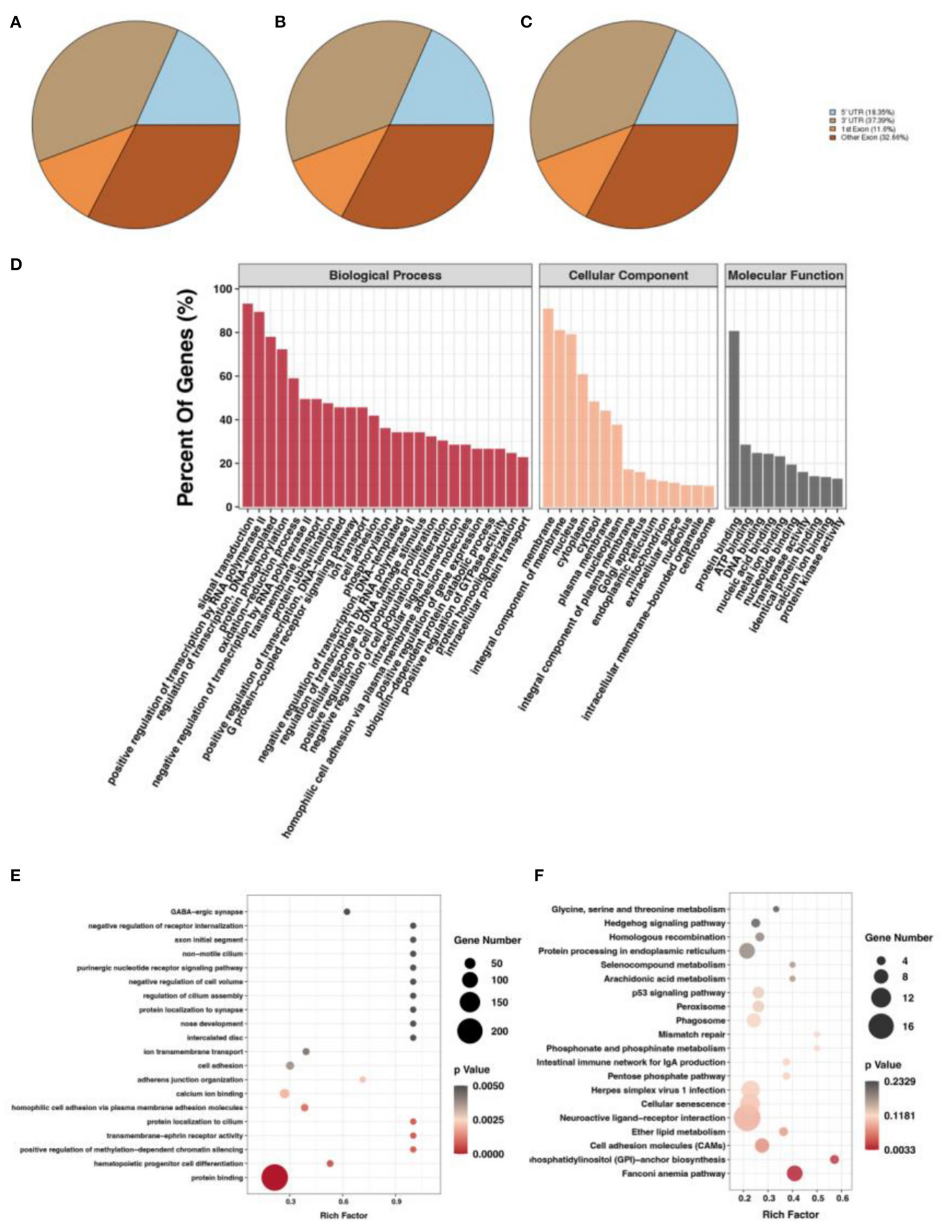
m6A methylation peak screening and differential m6A peak analysis. **(A)** m6A methylation peaks at E10. **(B)** m6A methylation peaks at E19. **(C)** Overlapped m6A methylation peaks between E10 and E19. **(D)** GO enrichment barplot of DMGs. **(E)** GO enrichment scatterplot of DMGs. **(F)** KEGG enrichment scatterplot of DMGs.

After screening for m6A methylation peaks, a total of 19,292 peaks were found at E10 (Supplementary Table S3), of which 8,538 were located in exons (44.26%), 7,213 in 3'UTR (37.39%), and only 3,541 in 5'UTR (18.35%; Figure 2A). In total, 11,294 genes were obtained with each of their transcripts overlapped with at least one m6A peak (called m6A genes in this study). For E19, a total of 16,843 peaks were found (Supplementary Table S4), of which 7,457 were located in exons (44.28%), 6,572 in 3'UTR (39.02%), and only 2,814 in 5'UTR (16.71%; Figure 2B). In total, 9,790 genes were obtained with each of their transcripts overlapped with at least one m6A peak. Differential analysis of the m6A methylated fragments from the two periods revealed that 6,774 differentially methylated peaks existed, of which 1,514 were highly expressed at E10 and 5,260 were lowly expressed at E10; meanwhile, 2,517 differentially methylated peaks were located in exons (37.15%), 2,588 in the 3'UTR (38.20%), and 1,669 in the 5'UTR (24.64%) (Figure 2C). These m6A differentially methylated peaks overlapped with 5,565 genes (DMGs) (Supplementary Table S5).

To investigate the function of DMGs in breast muscle development, we performed GO and KEGG enrichment analyses. GO term displayed DMGs were most significantly enriched in protein binding in molecular function, signal transduction in biological process, and membrane in cellular component, as shown in Figure 2D. In addition, many GO terms related to muscle development, fat deposition, and m6A methylation were also significantly enriched, including branchiomeric skeletal muscle development, RNA *N*^6^-methyladenosine methyltransferase complex, and fatty acid biosynthetic process (Figure 2E). KEGG results of DMGs are presented in Figure 2F. In total, we identified 123 pathways that were significantly enriched. Among them, the most significant pathways were neuroactive ligand–receptor interaction, cellular senescence, and cell adhesion molecules. Pathways related to muscle development and fat deposition were also significantly enriched, such as fatty acid elongation and phosphatidylinositol signaling system. We also identified the MYOG gene, a member of the MRFs (MyoD, MYOG, MyF5, and MRF4) family of myogenic regulators. The MYOG gene plays a key role in controlling the initiation of myoblast fusion, driving myoblast proliferation, and allowing the transition of mono-nucleated myoblasts into multinucleated myofibers (24).

### Motif analysis

m6A methylation modification, as well as demethylation modification, begins with the binding of multiple binding proteins to a motif at which methylation sites occur. Motifs are essentially nucleic acid sequence patterns of biological significance. m6A methylation and demethylation enzymes can recognize the motifs and bind to them, thereby affecting gene expression and gene function. The identification of these motifs is important for mechanistic studies of gene expression regulation. To determine whether m6A peaks contain a motif of m6A by RRACH (i.e., R for purine, A for m6A, C for cytosine, and H for non-guanine bases), we performed motif predictions for E10 and E19 samples (Supplementary Figure S2) and found motifs in both E10 and E19.

### Differentially expressed gene analysis

**Figure 3:**
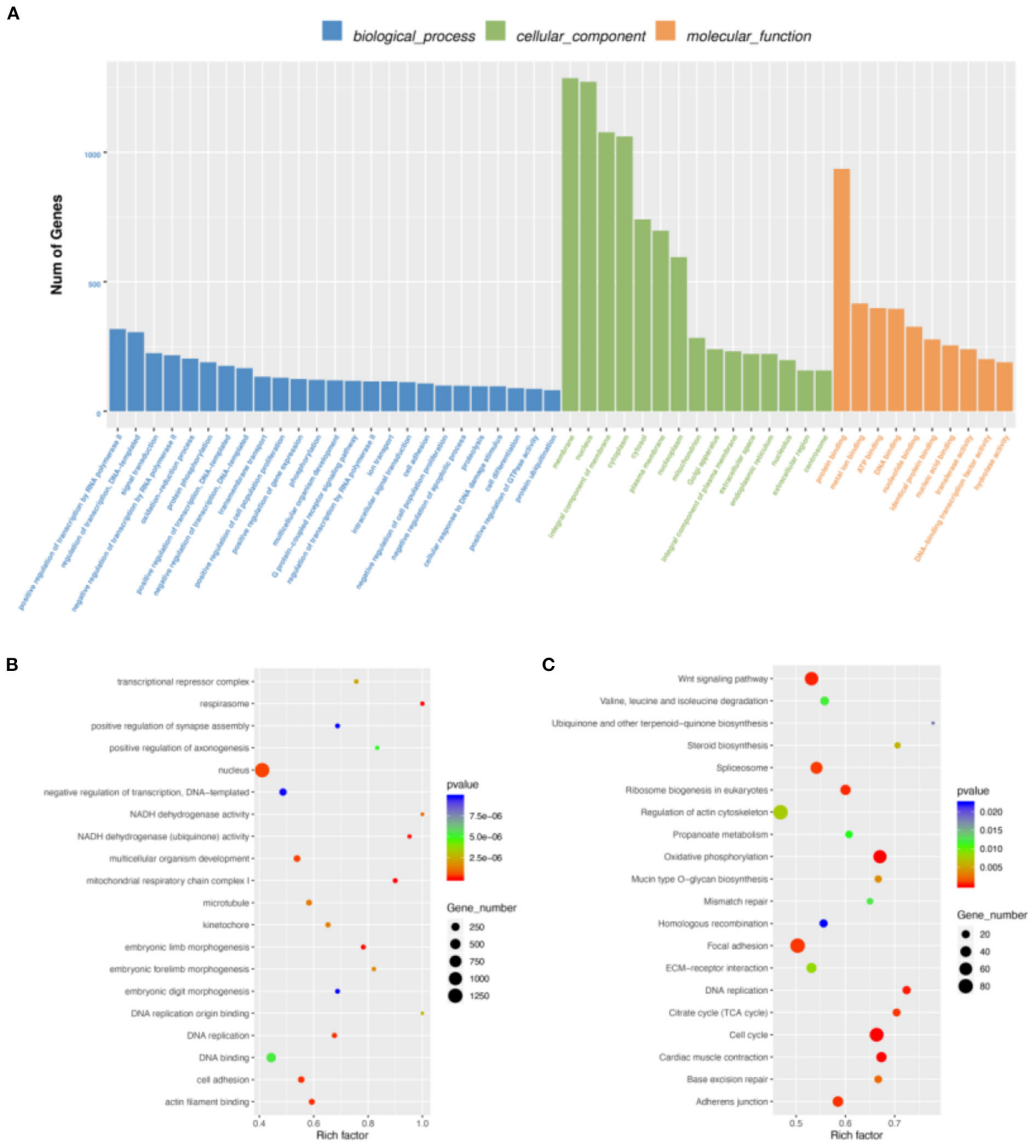
Differentially expressed gene (DEG) analysis. **(A)** GO enrichment barplot of DEGs. **(B)** GO enrichment scatterplot of DEGs. **(C)** KEGG enrichment scatterplot of DEGs.

By DEG analysis of RNA-seq data, we obtained 5,586 DEGs (Supplementary Table S6). GO and KEGG analyses were then performed. Biological process of GO analysis showed that positive regulation of transcription by RNA polymerase II was the most enriched. Molecular function of GO analysis indicated protein binding, while membrane was the most enriched in cellular component. (Figure 3A). More important, we found many muscle-related GO terms and genes (Figure 3B), such as those in the MRF family (the MYF6 gene was more highly expressed at E19 than at E10 and the MYOG Gene was more highly expressed at E10 than at E19) and Pax family (Pax3 and Pax7 were both more highly expressed at E10 than E19). Pax3 and Pax7 in the Pax family have DNA activating transcription factor activity and bind RNA polymerase II specifically; they also play an important role in myofiber cell development (25). These results further suggest that some genes in the MRF family and Pax family may function during breast muscle development of Wenchang chickens at different embryonic ages. KEGG enrichment analysis of the DEGs showed that 160 pathways were significantly enriched including cell cycle, oxidative phosphorylation, cardiac muscle contraction, and DNA replication (Figure 3C). Among them, some muscle development-related pathways, such as the Wnt signaling pathway, were also obtained (26).

### Conjoint analysis of DEGs and DMGs

**Figure 4:**
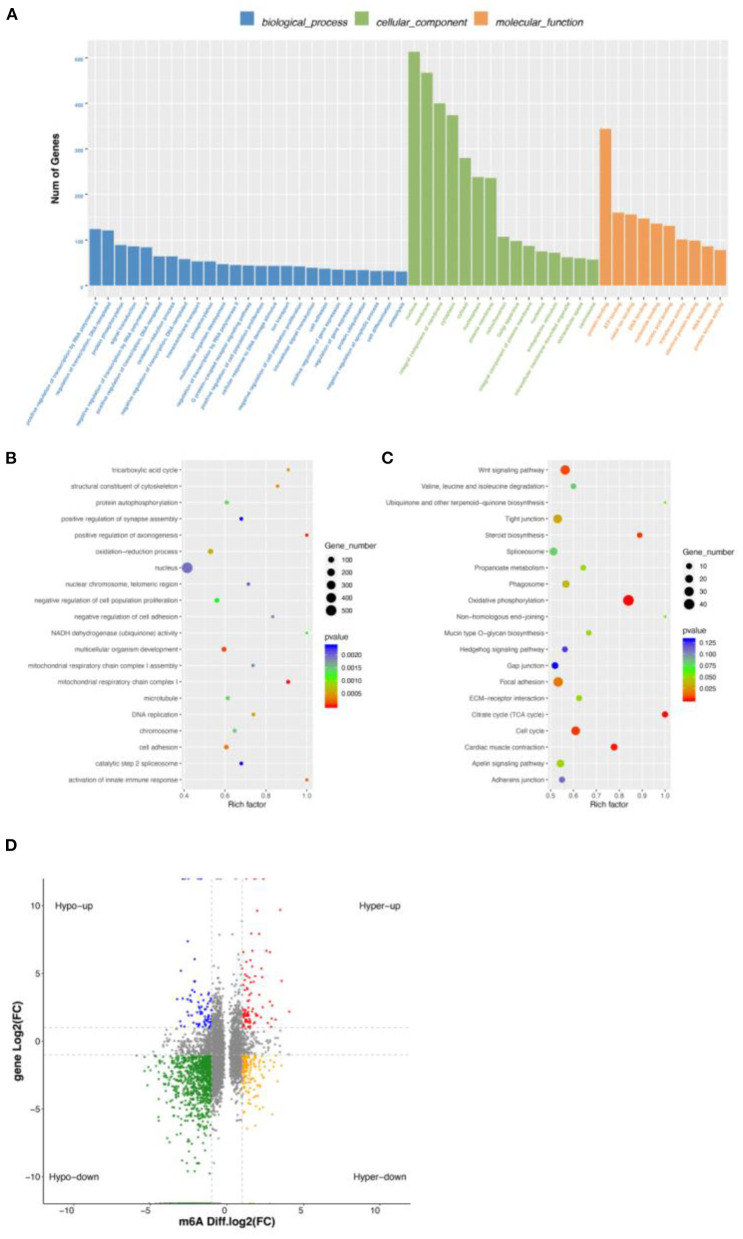
Conjoint analysis of DEGs and DMGs. **(A)** GO enrichment barplot of DEMGs. **(B)** GO enrichment scatterplot of DMGs. **(C)** KEGG enrichment scatterplot of DEMGs. **(D)** Regulatory relationship between m6A and differential genes.

To explore the relationship between m6A modification and gene expression, we carried out conjoint analysis of DEGs and DMGs. We overlapped DEGs and DMGs to produce 651 DEMGs in this study (Supplementary Table S7). GO functional analysis showed that most GO terms were enriched in cellular component (Figure 4A) and those DEMGs were significantly enriched in nucleus, positive regulation of transcription by RNA polymerase II, and protein binding (Figure 4B). KEGG results showed that 144 significantly enriched pathways were found, among which the most significant were Wnt signaling pathway, tight junction, and oxidative phosphorylation (Figure 4C). Interestingly, some muscle development-related pathways were also enriched, including the MAPK signaling pathway, an important regulatory pathway during myoblast differentiation (27). Activation of the MAPK signaling pathway can further increase skeletal myofiber cellular protein content, increase myofiber length and cross-sectional diameter, and make skeletal myofibers increase in mass when the number is unchanged (28).

We found a different correlation between methylated m6A level and gene expression abundance in E10 and E19 (Figure 4D). In 1,514 hyper-methylated m6A sites detected by m6A-seq, we found 314 genes with downregulated mRNA transcripts, that is, “hyper-down.” There were 187 genes detected to have hyper-methylated m6A sites along with upregulated mRNA transcripts, that is, “hyper-up.” In parallel to 5,260 hypo-methylated m6A sites, we found 214 genes with upregulated mRNA transcripts, that is “hypo-up.” There were 1,279 genes examined to have hypo-methylated m6A sites along with downregulated mRNA transcript, that is, “hypo-down.” It is easy to see that hypo-down m6A level and gene expression in E10 compared with E19 were higher than in others, but in our previous research on ducks and geese, most genes were enriched in hypo-up and hyper-down indicating that m6A showed a negative correlation. This may be because those genes are not only regulated by m6A modification, but also by other regulatory factors such as miRNA.

### miRNA analysis

Some previous research demonstrated that m6A could influence miRNA production (29), so we performed miRNA-seq to explore the effect. In total, 150 significantly differentially expressed miRNAs (DEMs) were obtained by miRNA-seq analysis. Among them, 58 were upregulated and 92 were downregulated (Supplementary Figure S3). The target genes of DEMs were predicted, and 5,675 target genes were obtained. Interestingly, some m6A key enzyme genes were found to be the targets of DEMs. For example, METTL14 was found to be the target gene of gga-miR-6586-5p and gga-miR-132a-3p; and YTHDF1 was the target gene of gga-miR-6555-5p and gga-miR-1626-5p. This suggests that miRNA may be involved in regulating m6A modification.

### Integrated analysis of m6A-seq, mRNA-seq, and miRNA-seq data

**Figure 5:**
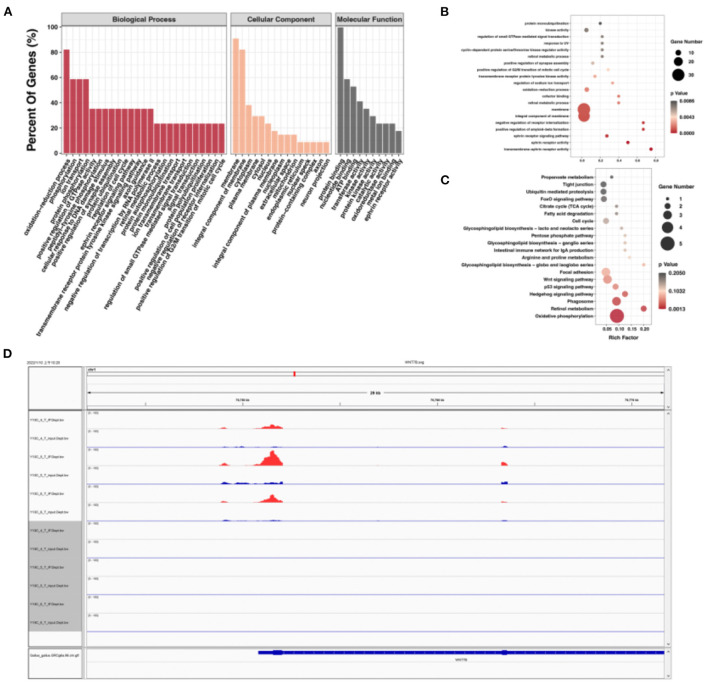
Integrated analysis of m6A-seq, mRNA-seq, and miRNA-seq data. **(A)** GO enrichment barplot of miRNA-m6A-genes. **(B)** GO enrichment scatterplot of miRNA-m6A-genes. **(C)** KEGG enrichment scatterplot of miRNA-m6A-genes. **(D)** The visualization of m6A abundances in *WNT7B* mRNA transcripts in E10 and E19.

Using overlapped DEMGs and the targets of DEMs, 72 miRNA-m6A-genes were obtained Supplementary Table S8. Then, GQ and KEGG functional analysis of miRNA-m6A-genes were performed. GO terms showed that they were significantly enriched in oxidation–reduction process, membrane, integral component of membrane, and cytoplasmic (Figure 5A). In addition, many GO terms were related to membrane, integral component of membrane, ephrin receptor activity, and others (Figure 5B). KEGG metabolic pathway enrichment analysis revealed that signal transduction, carbohydrate metabolism, lipid metabolism, glycan biosynthesis and metabolism, and amino acid metabolism were significantly enriched (Figure 5C). Among those miRNA-m6A-genes, we chose *WNT7B*, which is closely related with skeletal muscle development, to perform IGV visualization analysis and found significantly different m6A levels on the *WNT7B* gene between E10 and E19 (Figure 5D).

## Discussion

There are many types of modifications on RNA, including m6A, N^1^-methyladenosine modification (m1A), methylcytosine modification (m5C), and pseudouridine modification (Ψ). m6A was first discovered in the 1970s (30, 31); it is the most common internal modification in eukaryotic cell RNA and the best studied RNA modification. Studies have shown that m6A is common in mRNAs of different prokaryotes, eukaryotes, and viruses (32–34). m6A is the most common modification method in post-translational modification of eukaryotic mRNA, which accounts for more than 80% of RNA methylation (35). m6A is also a way for regulating mRNA metabolism and translation and plays important role in various physiological processes such as cell differentiation, development, and the stress response (2, 36).

m6A modification plays crucial role in many physiological processes such as myogenesis, embryonic development, and abnormal m6A modification can have huge effects on embryonic stem cell differentiation, zygote formation, and skeletal muscle development. Knockout of METTL3 in the embryonic stem cells before mouse embryo implantation will affect the stability of original pluripotency factor gene transcripts such as *Nanog* (homeobox protein NANOG), so that it cannot be downregulated, resulting in the obstruction of embryonic stem cell differentiation and the failure to fully terminate its naive state (37). Recently, another study has shown that the number of related gene transcripts cannot be changed during the development in oocytes of YTHDF2-messing mice; this phenomenon leads to infertility in this mouse (38). During embryonic development, muscle production is also inseparable from the regulation of m6A methylation. It has been shown that FTO as a demethylase of m6A can influence the differentiation of myoblasts. Deng et al. indicate that the FTO-mediated m6A modification in GADD45B mRNA drives skeletal muscle differentiation by activating the p38 MAPK pathway, which provides a molecular mechanism for the regulation of myogenesis via RNA methylation (39). Kudou et al. (40) found that METTL3 is involved in skeletal muscle differentiation of myogenic progenitor cells by mediating myogenic transcription factors such as MyoD, a key regulator of skeletal muscle differentiation. The results of our previous study illustrated the significance of m6A regulatory function in skeletal muscle development. Therefore, we selected breast muscles at E10 and E19 for preliminary experiments and found that m6A expression levels and methylation-modifying enzymes decreased with embryo age, indicating that m6A methylation modification played key role in the development of breast muscle in chicken embryos.

m6A methylation modification is a common phenomenon in post-transcriptional mRNA. Zhang et al. explored the expression profile of m6A in adult human tissues and detected a total of 101,340 methylation sites (41); the analysis of Dan et al. yielded 12,769 putative m6A sites within 6,990 coding gene transcripts and 250 noncoding ones (2). In this study, we found 18,068 peaks, which was consistent with the above studies. In addition, we found that the m6A methylation sites were mainly distributed in the 3'UTR regions, which are also the main binding sites of miRNAs. The m6A modification of the mRNA was mostly enriched near the terminators and in the 3′-UTR (36); it showed the same mode of distribution as our results.

We obtained 5,565 DMG overlapped peaks between E10 and E19, indicating that m6A modification was prevalent in chicken gene modification. Using RNA-seq, we identified 5,586 DEGs between E10 and E19. We overlapped DMGs with DEGs and noted 651 DEMGs that were significantly affected by development stages and m6A modification. GO and KEGG results show that many genes were enriched in skeletal muscle development and fat deposition. Our results illustrated that m6A methylation modification had important effects on skeletal muscle development and fat deposition.

In addition, we found many muscle development-related genes and m6A modification-related genes, such as MYH gene, MYF5 gene, Pax gene, METTL14 gene, YTHDF1 gene, and MYOG gene among DEMGs; this indicated that both development stages and m6A modification might be regulators of skeletal muscle in Wenchang chickens. The roles of the myogenic regulatory factor (MRF) family (MyoD, myf5, myogenin, and MRF4) can cause the activation, proliferation, and differentiation of satellite cells (24). Studies have shown that Pax3 and Pax7 are upstream transcription factors of the MRF genes, which can cause expression of the MyoD gene in the embryonic period, thereby affecting the occurrence of muscle cells (25). Pax3 mutant mice will have missing limb muscles, but precursor cells and body muscle (including thoracic muscle) show normal differentiation (42); in murine knockouts of both Pax3 and Pax7, skeletal muscle development will end in the embryonic stage (43). After comparing gene expression levels in Wenchang chicken E10 and E19 breast muscle tissues, we found that the expression levels of the Pax7 gene, Pax3 gene, METTL14 gene, YTHDF1 gene, and MYOG gene were lower in E19 than in E10. However, the expression levels of MYF6 and MYH genes were higher in E19 than that in E10. Considering the fact that the embryonic breast muscle growth of Wenchang chickens is slower at E19 than at E10, Pax7, Pax3, METTL14, YTHDF1, and MYOG genes might be positive regulators, while MYF6 and MYH genes may be negative regulators.

miRNAs are important regulatory factors in skeletal muscle. To detect their effect on the development of breast muscle, we combined them with the results of m6A-seq data, to find those genes regulated by both m6A modification and miRNAs. We first carried out miRNA-seq analysis of embryonic breast muscles of Wenchang chickens at E10 and E19. We obtained 495 DEMs and 5,676 target genes of DEMs. Using overlapping the targets of DEMs with DEMGs, we obtained 72 miRNA-m6A-genes, which might be the regulators of embryonic breast muscle development of Wenchang chickens with their expression regulated by miRNAs and m6A modification. Among those miRNA-m6A-genes, we chose *WNT7B*, which is closely related with skeletal muscle development, to perform IGV visualization analysis and found significantly different m6A levels on the *WNT7B* gene between E10 and E19.

There may be another regulation mode here, that is, m6A modification regulates the expression of miRNA and then affects the expression of mRNA. The pri-miRNA is often modified by m6A methyltransferase and cleaved by methylated binding proteins to become pre-miRNA and eventually forms mature miRNA. Alarcon et al. found that METTL3 can methylate pri-miRNA, and the labeled pri-miRNA can be recognized and processed by DGCR8. By miRNA chip analysis, it was found that knockdown of METTL3 would reduce the binding between DGCR8 and pri-miRNA, reduce the expression of mature miRNA, and increase the content of unprocessed pri-miRNA. *In vitro* experiments, it has been confirmed that m6A could promote the processing of pri-miRNA. Finally, functional validation experiments revealed that METTL3 could promote miRNA maturation (44). Kyung-Won et al. uncovered the role of mRNA methylation on the abundance of AGO2 mRNA resulting in the repression of miRNA expression during the process of human aging (45). m6A modification can not only directly regulate gene expression, but also indirectly affect related regulatory factors to affect gene expression. We therefore suggest that m6A modification is important and extensive in organisms; however, the related regulatory mechanism remains to be studied.

## Conclusion

In conclusion, we compared m6A modification profiles and gene expression levels at E10 and E19 of Wenchang chickens. We found that some genes shared by DMGs and DEMs in chicken embryonic breast muscle were enriched in skeletal muscle development-related pathways, indicating that m6A modification is one approach affecting skeletal muscle development and fat deposition. In addition, some target genes of DEMs overlapped with DEMGs, which indicated that miRNAs were potential regulators of m6A modification.

## Data availability statement

The datasets presented in this study can be found in online repositories. The names of the repository/repositories and accession number(s) can be found below: Sequence Read Archive (SRA); PRJNA836372 and PRJNA835987.

## Ethics statement

The animal study was reviewed and approved by Institute of Animal Science & Veterinary Medicine, Hainan Academy of Agricultural Sciences.

## Author contributions

LG, TX, and HZ conceived and designed the experiments. LG, YC, XZ, and TX performed the experiments. TX and QJ analyzed the data. TX, LG, and QJ wrote the paper. All authors read and approved the final manuscript.

## Funding

This study was supported in part by the Key R&D projects in Hainan Province (SQ2020XDNY0161), the National Key R&D Program of China (Grant No. 2021YFD1300100), and the Project of Wenchang Chickens Research Institute (2,426), Basal Research Fund for Hainan Academy of Agricultural Sciences (JBKYYMF-2020-09).

## Conflict of interest

The authors declare that the research was conducted in the absence of any commercial or financial relationships that could be construed as a potential conflict of interest.

## Publisher's note

All claims expressed in this article are solely those of the authors and do not necessarily represent those of their affiliated organizations, or those of the publisher, the editors and the reviewers. Any product that may be evaluated in this article, or claim that may be made by its manufacturer, is not guaranteed or endorsed by the publisher.

## Supplementary material

Supplementary Table S1 Basic information of sequenced data.

Supplementary Table S2 Mapping summary of chicken reference genome.

Supplementary Figure S1 Peak distribution of Wenchang chickens in E10 and E19.

Supplementary Table S3 m6A methylation peaks in E10.

Supplementary Table S4 m6A methylation peaks in E10.

Supplementary Table S5 Differentially methylated genes (DMGs) in E10 and E19.

Supplementary Figure S2 Motif sequence of m6A contained.

Supplementary Table S6 Differentially expressed genes (DEGs) in E10 and E19.

Supplementary Table S7 Shared genes by overlapped DEGs and DMGs, (DEMGs) in E10 and E19.

Supplementary Figure S3 Significantly differentially expressed miRNAs (DEMs) in E10 and E19.

Supplementary Table S8 miRNA-m6A-genes in E10 and E19.

## Abbreviations

DMGs: differentially methylated genes
DEGs: differentially expressed genes
DEMGs: shared genes between DEGs and DMGs
input: the data of RNA-seq
IP: the date of m6A-seq.
