# Supplementary material for: Transcriptome-wide study revealed m6A and miRNA regulation of embryonic breast muscle development in Wenchang chickens
Source: Front Vet Sci. 2022 Jul 26;9:934728. doi: 10.3389/fvets.2022.934728 (PMC9360417; doi:10.3389/fvets.2022.934728)
Supplement: Supplementary Table S1 — Basic information of sequenced data. [file Table_1.DOCX]

Table 1. Basic information of sequenced data

| Sample_ID | Raw_Reads | Raw_Bases | Valid_Reads | Valid_Bases | Valid% | Q20% | Q30% | GC% |
| --- | --- | --- | --- | --- | --- | --- | --- | --- |
| E10_IP-1 | 72344140 | 10.85G | 53452148 | 7.45G | 68.68 | 95.66 | 89.32 | 50.44 |
| E10_IP-2 | 75011998 | 11.25G | 66010820 | 9.22G | 81.91 | 95.49 | 89.03 | 50.66 |
| E10_IP-3 | 65374768 | 9.81G | 45455908 | 6.28G | 64.00 | 96.83 | 91.76 | 51.50 |
| E19_IP-1 | 70391726 | 10.56G | 56143180 | 7.77G | 73.55 | 96.35 | 90.65 | 50.36 |
| E19_IP-2 | 70279666 | 10.54G | 47513166 | 6.54G | 62.06 | 96.28 | 90.59 | 50.75 |
| E19_IP-3 | 73724372 | 11.06G | 42567424 | 5.76G | 52.05 | 96.55 | 91.14 | 51.11 |
| E10_input-1 | 69553556 | 10.43G | 51533886 | 7.06G | 67.69 | 97.21 | 92.31 | 50.42 |
| E10_input-2 | 62517854 | 9.38G | 49855050 | 6.88G | 73.36 | 97.13 | 92.12 | 50.35 |
| E10_input-3 | 70848156 | 10.63G | 51015808 | 7.01G | 65.93 | 97.23 | 92.33 | 51.16 |
| E19_input-1 | 71238812 | 10.69G | 49673474 | 6.79G | 63.56 | 97.26 | 92.40 | 50.75 |
| E19_input-2 | 63338554 | 9.50G | 49118654 | 6.81G | 71.64 | 97.04 | 91.75 | 51.04 |
| E19_input-3 | 66031952 | 9.90G | 51200524 | 7.10G | 71.71 | 96.97 | 91.59 | 50.77 |

Note: input represents the data of RNA-seq, IP represents the date of m6A-seq.
