# Supplementary figures and images for: Transcriptome-wide study revealed m6A and miRNA regulation of embryonic breast muscle development in Wenchang chickens
Source: Front Vet Sci. 2022 Jul 26;9:934728. doi: 10.3389/fvets.2022.934728 (PMC9360417; doi:10.3389/fvets.2022.934728)

E10-1-IP

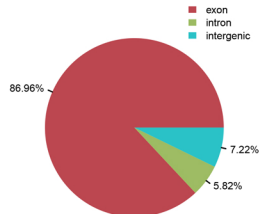

E10-2-IP

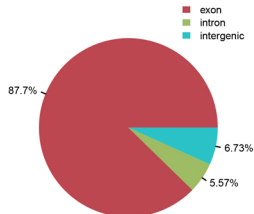

E10-3-IP

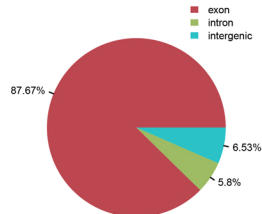

E19-1-IP

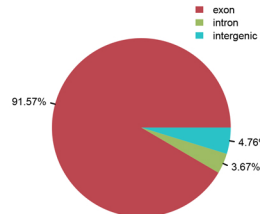

E19-2-IP

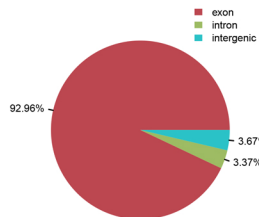

E19-3-IP

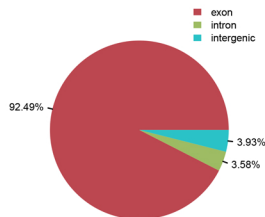

E10-1-input

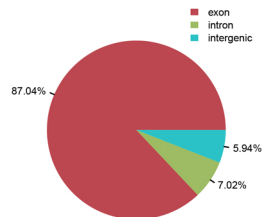

E10-2-input

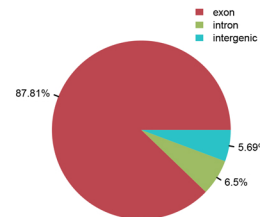

E10-3-input

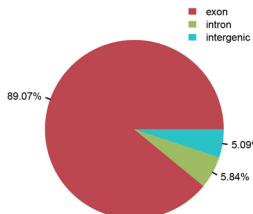

E19-1-input

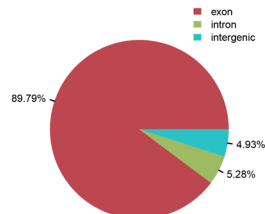

E19-2-input

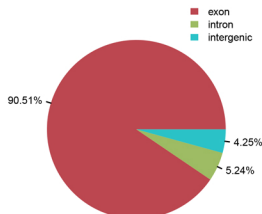

E19-3-input

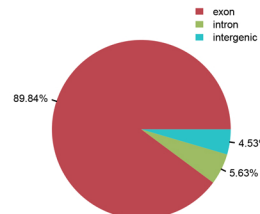

Supplement: Supplementary Figure S1 — Peak distribution of Wenchang chickens in E10 and E19. [file Image_1.PDF]

Bits

0.8

0.6

0.4

0.2

0.0

1

2

3

4

5

6

7

8

9

10

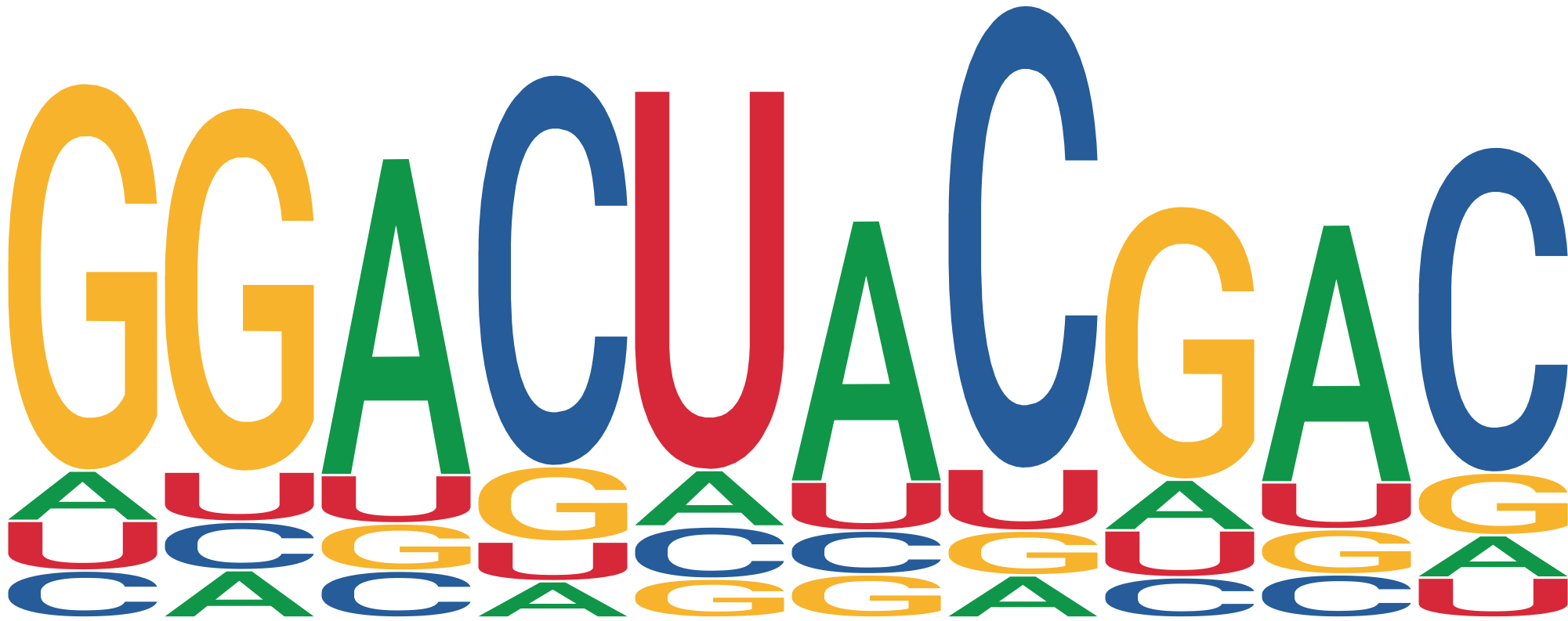

Supplement: Supplementary Figure S2 — Motif sequence of m6A contained. [file Image_2.PDF]

# Differentially expressed miRNAs in different groups

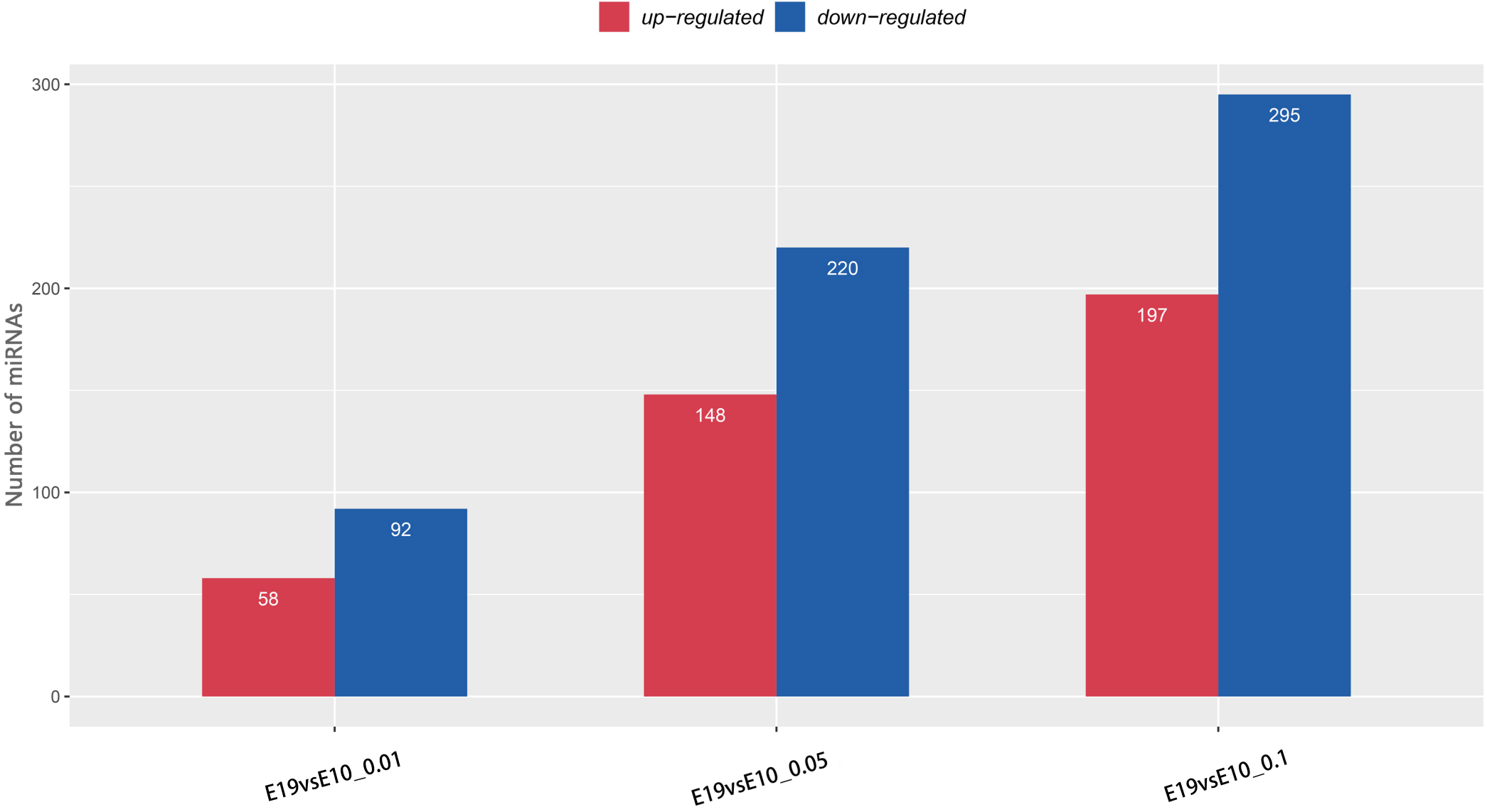

Supplement: Supplementary Figure S3 — Significantly differentially expressed miRNAs (DEMs) in E10 and E19. [file Image_3.PDF]
